# Infective endocarditis; report from a main referral teaching hospital in Iran

**Published:** 2017

**Authors:** Behrooz Heydari, Iman Karimzadeh, Hossein Khalili, Esfandiar Shojaei, Abdolrasool Ebrahimi

**Affiliations:** a *Depatrment of Clinical Pharmacy, Faculty of Pharmacy, Shahid Sadoughi University of Medical Sciences, Yazd, Iran. *; b *Depatrment of Clinical Pharmacy, Faculty of Pharmacy, Shiraz University of Medical Sciences, Shiraz, Iran. *; c *Depatrment of Clinical Pharmacy, Faculty of Pharmacy, Tehran University of Medical Sciences, Tehran, Iran. *; d *Depatrment of Infectious Diseases, Faculty of Medicine, Tehran University of Medical Sciences, Tehran, Iran.*; e *Petroleum Industry Health Organization, Asalouyeh, Iran.*

**Keywords:** Infective endocarditis, clinical presentation, treatment, outcome

## Abstract

**Background/Objective:**

The aim of the present preliminary study was to assess the demographic, clinical, paraclinical, microbiological, echocardiographic, and therapeutic profile as well as in-hospital outcome of patients with infective endocarditis at a referral center for various infectious diseases in Iran.

**Methods:**

Required demographic, clinical, plausible complications and paraclinical data were collected from patients’ medical charts. Echocardiographic findings were obtained by performing transthoracic and/or transesophageal echocardiography as clinically indicated. In addition, details of management modalities and in-hospital outcome of patients were recorded.

**Results:**

During a 3-year period, 55 patients with definite or possible diagnosis of Infective endocarditis were admitted to the ward. Twenty one (38.2%) patients were injection drug users. *Staphylococcus aureus* and *S.epidermidis* were the most commonly isolated microorganisms. Management modalities of Infective endocarditis included antimicrobial therapy alone (48 cases) and the combination of antimicrobial therapy and surgery (7 cases).

**Conclusion:**

The rate of negative blood culture in our cohort is high. *S. aureus* and *S.epidermidis* were the most commonly isolated microorganisms from positive blood cultures. Congestive heart failure was the most frequent infective endocarditis complication as well as indication for surgery. In-hospital mortality rate of patients was unexpectedly low.

## Introduction

Infective endocarditis (IE) is an infection of either the healthy or injured heart valves or its inner lining (mural endocardium) caused by infectious agents such as bacteria, fungi, and possibly viruses (1). In the United States and Western Europe, the incidence of community-acquired native-valve IE in most recent studies is 3 to 10 cases per 100,000 person-years (2, 3). Despite considerable advances in diagnosis techniques and treatment modalities, it remains as a serious and life-threatening infection with relatively high morbidity and mortality rates (4, 5) ranges from 16% to 25% of affected individuals (6).

In developed countries, increase in life expectancy of patients, emergence of new predisposing factors, and high prevalence of nosocomial cases has led to considerable changes in the epidemiological pattern of IE (7-9). Studies about the profile of IE in developing countries are relatively limited and scarce and many aspects of IE in these regions remain unknown. These data can be exploited by clinicians to develop optimal diagnostic, preventive, and therapeutic strategies for this complex clinical setting. The aim of the present preliminary study was to assess the demographic, clinical, paraclinical, microbiological, echocardiographic, and therapeutic profile as well as in-hospital outcome of patients with IE at a referral center for various infectious diseases in Iran. To the best of our knowledge, this topic has not been considered comprehensively in relevant epidemiological studies in our country so far.

*Methods*

A retrospective study was conducted on medical records of all adult (> 18 years) individuals with definite or possible diagnosis of IE admitted to 60-bed infectious diseases ward of Imam Khomeini Hospital, a multispecialty, tertiary, healthcare university setting affiliated to Tehran University of Medical Sciences, Tehran, Iran from 2007 to 2010. Diagnosis of IE was based on the modified Duke’s criteria (10).

Required demographic (age, sex), clinical (underlying heart disease, history of intravenous drug abuse, co-infections and other co-morbidities, relevant constitutional manifestations [e.g. fever, night sweats, weight loss, Osler nodes, Janeway lesions, Roth spot, Splinter hemorrhage, splenomegaly], plausible complications [e.g. heart failure, acute renal failure, thromboembolic events, intracranial hemorrhage]), and paraclinical (hemoglobin level, platelet count, leukocyte count, erythrocyte sedimentation rate, serum creatinine) data were collected from patients’ medical charts. Echocardiographic findings including site of infection (e.g. mitral, tricuspid), type of affected heart valve (native versus prosthetic), and valvular complications (e.g. perforation, abscess, dehiscence, moderate or severe regurgitation) were obtained by performing transthoracic (TTE) and/or transesophageal (TEE) echocardiography as clinically indicated. Standard methods including gram staining, biochemical tests, and Kirby-Bauer disc diffusion were used to identify causative microorganisms (bacteria or fungi) as well as determine their antimicrobial susceptibility profile from collected blood samples before starting antimicrobial treatment. In addition, details of management modalities including medical (antibiotic regimen, duration) and/or surgery (indication and type of intervention) treatment were recorded. In-hospital outcome of patients including discharge or death was also gathered. 

*Statistical analysis*

Continuous variables were expressed as means ± standard deviation (SD) and categorical data as percentages. Descriptive analyses were performed by the Statistical Package for the Social Sciences (SPSS) version 15 (SPSS Inc., Chicago, IL, USA). 

## Results

During a 3-year period, 55 patients with definite or possible diagnosis of IE were admitted to the ward. Demographic, clinical, paraclinical, and echocardiographic characteristics of the study population are demonstrated in Table 1. More than three-fourth (78.2%) of them were males. Twenty one (38.2%) patients were injection drug users (IDU). Rheumatic heart disease (RHD, 7 cases) and congenital heart diseases (CHD, 5 cases) were the most frequent underlying heart diseases in the study population. Fever, defined by either oral temperature above 37.2 or 37.8 Celsius degrees in the morning and afternoon, respectively,was the most common clinical symptom (90.9%) followed by chills (69.1%), weight loss (45.5%), and night sweating (41.8%). Their mean ± SD temperature at ward admission was 38.7 ± 0.82 Celsius degrees. Splinter hemorrhage and Roth spots was detected in 3 (5.5%) and 2 (3.6%) individuals, respectively. In addition, each Osler›s nodes and Janeway lesions were observed only in 1 (1.8%) subject. Regarding related laboratory findings, normocytic normochromic anemia (hemoglobin level < 12 g/dL in females and < 13 g/dL in males), elevated erythrocyte sedimentation rate (ESR > 7 mm/h for those < 40 years of age and > 20 mm/h for > 40 years of age), and leukocytosis (leukocyte count > 11,000/µL) were detected in 87.3%, 61.8%, and 40% of individuals, respectively. IE developed on a native and mechanical prosthetic heart valve in 80% and 20% of patients, respectively. Left valves of the heart were more affected than the right ones. The mitral valve was the most frequent involved valve (33 cases) followed by tricuspid (15 cases). However among IDU patients, tricuspid valve (71.4%) was the predominant infected heart valve. Moderate or severe valvular regurgitation and abscess were detected in 6 (10.9%) and 1 (1.8%) individuals, respectively.

Only blood cultures of 17 (30.9%) patients were positive before starting antimicrobial treatment. In other words, the rate of negative blood culture was 69.1%. *Staphylococcus aureus* (35.3%) and *S.epidermidis* (17.6%) were the most commonly isolated microorganisms both comprising 52.9% of all positive blood cultures. More than fifty percent (54.5%) of *S. aureus *isolates were resistant to methicillin (MRSA). Two isolates of MRSA were also resistant to vancomycin (VRSA). After *Staphylococci sp.*,* Streptococci sp. (viridans&pneumoniae)* and Enterococciaccount for 17.7% and 11.8% of all positive blood cultures, respectively. All isolates of *Streptococci sp. *were susceptible to vancomycin and ceftriaxone. In addition, both Enterococci isolates were sensitive to ampicillin, gentamicin, and vancomycin. The distribution of causative microorganisms isolated from the blood culture of the study population is listed in Table 2.

Management modalities of IE included antimicrobial therapy alone (48 cases) and the combination of antimicrobial therapy and surgery (7 cases). Congestive heart failure was the most common indication for surgery. The 3 most frequent antimicrobial combination regimen were ampicillin plus cloxacillin plus gentamicin (67.3%), cloxacillin plus gentamicin (7.3%), and ceftriaxone plus gentamicin (5.5%) (Table 3). Two individuals with isolated MRSA resistant to vancomycin were treated successfully by a 6-week course of linezolid therapy alone or in combination with rifampin without any episodes of bone marrow suppression such as thrombocytopenia. The mean ± SD length of antimicrobial therapy was 12.85 ± 7.8 days (range, 3-42 days). In addition, the mean ± SD number of antimicrobials administered within the ward stay was 3.38 ± 1.63 (range, 1-8). 

Regarding IE complications, heart failure, thromboembolic events, and acute renal failure requiring temporary hemodialysis occurred in 8 (14.55%), 6 (10.91%), and 2 (3.64%) individuals, respectively. Four (7.3%) patients died during the ward stay and the remaining 51 (92.7%) discharged from the hospital.

## Discussion

The mean ± SD age of our cohort (33.9 ± 1.2 years) is similar to that reported from developing countries (11-13) but considerably less than that in developed areas (56 ± 17 years)(14). These differences can be partially justified by the fact that RHD is the major underlying predisposing heart disease in developing countries (1, 2). In line with this, RHD was the most frequent underlying heart disease in our study population. In contrast, RHD prevalence has decreased continuously along with a counterpart increased in the prevalence of degenerative heart diseases^5^and the rate of using invasive procedures in Western countries (15). In accordance with other relevant studies, male were considerably more affected with IE than females (78.2% versus 21.8%, respectively) with male-to-female ratio of 3.6:1. This ratio in relevant literature has been reported to range from 3:2 to 9:1 (16-18). Our results regarding clinical and paraclinical features of the patients such as fever and anemia are also comparable with other epidemiological investigations in the field of IE from both developed and developing countries (19-21).

Similar to many relevant studies in patients with IE in developing countries (13, 19, 22), native and mitral valve were the most common type (80%) and site (60%) of infected heart valve in the present study, respectively. Currently, prosthetic valve endocarditis accounts for about 7-25% of all cases of IE in most developed countries (23). However, it has been speculated that increase in access to medical facilities and the number of heart valve implantations may enhance the proportion and significance of prosthetic valve IE in the near future (24). Regarding site of infection, there is a strong association between IDU and involvement of right-sided heart valves with IE (2). In this regards, Besharat *et al*. in a descriptive study on 33 Iranian IDU patients with IE in 2 tertiary teaching hospitals in Tehran during 2002-2008 reported tricuspid as the most frequent involved heart valve (45%) followed by aortic (15%), and mitral (5%) (25). In our survey, less than two-fifth (38.2%) of subjects were IDUs and the rate of right-sided heart valve involvement was above 2.5 times lower than that of left-sided (72.7% versus 27.3%, respectively). Nevertheless within IDUs in the current study, tricuspid was the most frequent involved heart valve which is in accordance with the relevant literature. 

The percentage of negative blood culture in our study (69.1%) is far beyond the 10% rate reported in recent publications from developed countries (5,26). Our data is also higher than that reported from other developing countries such as Brazil (35%) (19), Turkey (36.1%) (22), India (59%) (27),and Tunisia (49%) (28). The rate of culture-negative IE in Besharat *et al*. study was 43%. In addition, 30% of their patients had no blood culture report in their medical charts (25). High prevalence of culture-negative IE may be attributed to inopportune previous administration of antibiotics, inadequate techniques of microbiological culturing, and involvement of highly fastidious bacteria (e.g. HACEK group)(29, 30). Regarding the first issue for example, the National Center of Rational Use Drug reported that antibiotics (e.g. oral amoxicillin) are among the 5 most commonly prescribed medications during years 2008 to 2010 in Iran (31). Negative blood culture IE can complicate diagnosis as well as optimal treatment course through selecting unnecessary or non-effective antibiotics which potentially can lead to antimicrobial resistance or adverse reactions (27). In this regards since the majority of blood cultures in our cohort were negative and the causative microorganism is unknown, it is not surprising that ampicillin plus cloxacillin plus gentamicin is the most common antimicrobial combination regimen (37 cases) to cover both *Staphylococci sp* and* Streptococci sp.*

*Staphylococci sp* along with* Streptococci sp. *are the major causative microorganism among patients with positive blood culture in our study. However, it is noteworthy that near 70% of the study population has negative blood cultures at diagnosis and the real pattern of causative microorganisms in our cohort may be somewhat different. Two prominent studies by Hoen *et al.* (26) and Fowler *et al.* (32) have reported similar findings. Besharat *et al.* were also reported *S. aureus *as the most frequent isolated microorganism from their IDU patients with IE (25). Their finding is expected because *S. aureus *is the most common infecting organism in IDUs (2, 3). It seems that *Staphylococci sp.*are surpassing *Streptococci sp. *asthe most common infecting agent in IE during these recent years (19, 22). This might be due to change in the spectrum of IE which is tend to occur more in older populations and at health-care context secondary to increase in access to medical facilities and invasive procedures (19).

More than fifty percent (54.5%) of isolated* S. aureus *from blood samples of our cohort were identified as MRSA. The rate of MRSA has increased from 2.4% in 1975 to 29% in 1991 in the US (33) and from 4% in 1990 to 42% in 2000 in the England and Wales (34). These rates in the Netherlands and Scandinavian countries are about 2% (35). In congruent with this ascending trend, our previous retrospective study in the same ward implicated that the percentage of MRSA has increased from 60.78% to 72% during a 4-year period from 2007 to 2010 (36). The probable risk factors of MRSA development such as prior antibiotic use, prolonged hospitalization, and hemodialysis were not assessed in the current study. Two cases of MRSA in our cohort were also resistant to vancomycin based on the disc diffusion method. There are only few real isolates of VRSA reported so far worldwide [at least 7 isolates from the US (37) and 1 isolate from India (38)].Aligholi *et al.* reported 2 strains of VRSA confirmed by the microbroth dilution and polymerase chain reaction methods at the same setting (Imam Khomeini hospital) over a period of 1 year in 2005 (39). It has been suggested that VRSA appears not to be a major concern in the antimicrobial resistance in the near future (40). Clinical responsiveness of our 2 detected VRSA isolates to linezolid treatment (alone or in combination with rifampin) was in accordance with reported susceptibility pattern of gram-positive bacteria to this agent in most studies from Iran (36). Despite very uncommon, experimental models, case reports, and clinical outbreaks of *S. aureus* resistance to linezolid has been described (41, 42). Due to the association of higher mortality with linezolid therapy in patients with catheter-associated bloodstream infections caused by gram-negative bacteria, potential serious adverse reactions (e.g. myelosuppression, peripheral neuropathy, lactic acidosis), and antimicrobial resistance, it should be used only under specific and selected circumstances (43).

Only 2 bacterial isolates from blood in our cohort (11.8%) were identified as Enterococci which both were sensitive to ampicillin, gentamicin, and vancomycin. However, the species of detected Enterococci (faecalis versus faecium) were undetermined. In the US, Enterococcus species account for approximately 12 % of all hospital-acquired infections. In Europe, Enterococci have considered as the third most common cause of bacteraemia. Five to 18% of all IE cases have been attributed to Enterococci species (44).Gharouni *et al.* in 2006 reported a case series of 11 individuals with Enterococcal IE in a teaching hospital in Tehran, Iran. Ten out of 11 subjects in their study responded to the combination of ampicillin and gentamicin and the remaining 1 patient resistant to ampicillin was treated successfully with vancomycin and gentamicin regimen for 6 weeks (45). According to results of our previous study from the same ward, the rates of ampicillin- and gentamicin-resistant Enterococci during a 4-year period (2007 to 2010) ranged from 14-60% and 25-67%, respectively (36).

**Table 1:** Demographic, clinical, paraclinical, and echocardiographic characteristics of the study population (n = 55

**Parameter**	**n (%)**
Gender	
Male	43 (78.2)
Female	12 (21.8)
Age (years)	
Mean ± SD	33.9 ± 1.2
Range	20-78
Concomitant infectious diseases	
HIV	14 (25.5)
HCV	10 (18.2)
TB	3 (5.5)
Underlying heart diseases	
Rheumatic heart disease	7 (12.7)
Congenital heart diseases	5 (9.1)
Pacemaker	2 (3.6)
Related clinical signs and symptoms	
Fever	50 (90.9)
Chills	38 (69.1)
Anemia	48 (87.3)
Weight loss	25 (45.5)
Night sweating	23 (41.8)
Peripheral edema	17 (30.9)
Dyspnea	11 (20)
Petechia	6 (10.9)
Splinter hemorrhage	3 (5.5)
Roth spots	2 (3.6)
Osler's nodes	1 (1.8)
Janeway lesions	1 (1.8)
Related paraclinical findings	
Anemia	48 (87.3)
Elevated erythrocyte sedimentation rate	34 (61.8)
Leukocytosis	22 (40)
Thrombocytopenia	14 (25.5)
Splenomegaly	7 (12.7)
New heart murmur	5 (9.1)
Type of involved heart valve	
Native	44 (80)
Mechanical prosthetic	11 (20)
Site of infection	
Mitral	33 (60)
Tricuspid	15 (27.3)
Aortic	5 (9.1)
Combined mitral and aortic	2 (3.6)

**Table 2:** Microbiological profile of patients with positive blood cultures (n = 17)

**Microorganism**	**n (%)**
*Staphylococcus aureus*	6 (35.3)
*Staphylococcus epidermidis*	3 (17.6)
*Streptococcus viridans*	2 (11.8)
Enterococci	2 (11.8)
*Streptococcus pneumoniae*	1 (5.9)
*Acinetobacterspp*	1 (5.9)
Enterobacter	1 (5.9)
Brucella	1 (5.9)

**Table 3:** Antimicrobial treatment regimens of the study population (n = 55)

**Antimicrobial regimen**	**n (%)**
Ampicillin plus cloxacillin plus gentamicin	37 (67.3)
Cloxacillin plus gentamicin	4 (7.3)
Ceftriaxone plus gentamicin	3 (5.5)
Vancomycin plus gentamicin	2 (7.3)
Ampicillin plus gentamicin	2 (3.6)
Vancomycin plus gentamicin plus rifampin	2 (3.6)
Imipenem plus gentamicin	1 (1.8)
Imipenem plus ciprofloxacin	1 (1.8)
Doxycycline plus rifampin plus gentamicin	1 (1.8)
Linezolid plus rifampin	1 (1.8)
Linezolid	1 (1.8)

The percentage of our cohort underwent heart valve surgery to manage IE (12.7%) is much lower than that reported in other similar studies from both developed and developing areas such as 23.1% in Indian (13), 26.2% in Argentinean (20), 49.7% (26) and 54% (46) in France, 50.7% in Tunisia (28), and 53% in Brazil (19). In contrast to the above data, in Math *et al.* prospective study between 2004 and 2006 from India, heart valve surgery was only done in 15% of individuals with IE (27).The plausible justifications for this finding in our as well as Math *et al* survey may be limited availability and affordability of heart valve surgery, hesitancy of cardiologist or cardiac surgeons about performing early cardiac surgery, and less severity and complexity of IE in our cohort than other studies. In confirming this last issue, in-hospital mortality rate in our study (7.3%) was much lower than that reported from the above investigations ranged from 19% to 31% (13, 19, 20, 26, 28, 46).Congestive heart failure as the major indication of cardiac surgery in the present survey is in accordance to literature data in this area (19, 28).

The present study has a number of limitations and our data should be interpreted with caution. First, although it was conducted on medical records over a 4-year period, the number of studied patients is relatively low in comparison to similar investigations from other parts of the world. Second, as included patients were from a tertiary referral center, our findings are inevitably vulnerable to center and selection bias and they may not be a real representative of IE even in our population in Iran. Third, since only 4 (7.3%) individuals with IE died during the ward (hospital) stay, it was not statistically feasible to evaluate predictive factors of in-hospital mortality which may require for risk stratification of patients and determining the optimal management modality. 

In conclusion, results of our preliminary retrospective study in Iran demonstrated that RHD continues to be the major underlying predisposing heart disease in patients with IE. The rate of negative blood culture in our cohort is high. *S. aureus* and *S.epidermidis* were the most commonly isolated microorganisms from positive blood cultures. Congestive heart failure was the most frequent IE complication as well as indication for surgery. Most of patients with IE were managed successfully with antimicrobial therapy alone. In-hospital mortality rate of patients was unexpectedly low. Developing a national database using the data of the current studyalong with performing large, multi-center, prospective studies can considerably assist health-care professionals and policy makers in monitoring probable changes in the epidemiological, microbiological, antimicrobial resistance, and clinical outcome pattern of IE in our country. Treatment guideline of IE can be also nationalized and modified based on these findings.

## Founding:

The authors have not received any found about this work.
